# The maternal and neonatal outcomes for an urban Indigenous population compared with their non-Indigenous counterparts and a trend analysis over four triennia

**DOI:** 10.1186/1471-2393-13-167

**Published:** 2013-08-30

**Authors:** Sue Kildea, Helen Stapleton, Rebecca Murphy, Machellee Kosiak, Kristen Gibbons

**Affiliations:** 1 Australian Catholic University, 1100 Nudgee Road, Banyo, QLD 4014, Australia; 2 Mater Research, Level 1, Aubigny Place, Raymond Terrace, South Brisbane, QLD 4102, Australia; 3 School of Nursing & Midwifery, Trinity College, 24 D'Olier Street, Dublin 2, Ireland

**Keywords:** Aboriginal and Torres Strait Islander, Indigenous Australian, Antenatal, Maternity, Midwifery, Culturally responsive, Model of care, Evaluation

## Abstract

**Background:**

Indigenous Australians experience significantly disproportionate poorer health outcomes compared to their non-Indigenous counterparts. Despite the recognised importance of maternal infant health (MIH), there is surprisingly little empirical research to guide service redesign that successfully addresses the disparities. This paper reports on a service evaluation that also compared key MIH indicators for Indigenous and non-Indigenous mothers and babies over a 12-year period 1998–2009.

**Methods:**

Trend analysis with logistic regression, using the independent variables of ethnicity and triennia, explored changes over time (1998–2009) between two cohorts: 1,523 births to Indigenous mothers and 43,693 births to non-Indigenous mothers. We included bivariate and multivariate analysis on key indicators (e.g. teenage births, preterm birth, low birth weight, smoking) and report odds ratios (ORs), 95% CIs and logistic regression adjusting for important confounders. We excluded transfers in from other areas which are identified within the database.

**Results:**

Bivariate analysis revealed Indigenous women were statistically more likely to have spontaneous onset of labour and a non-instrumental vaginal birth. They were less likely to take epidurals for pain relief in labour, have assisted births, caesarean sections or perineal trauma. Despite better labour outcomes, Indigenous babies were more likely to be born preterm (< 37 weeks) and be low birth weight (< 2500 g); these differences remained significant in multivariate analysis. The trend analysis revealed relatively stable rates for teenage pregnancy, small for gestational age, low birth weight babies, and perinatal mortality for both cohorts, with the gap between cohorts consistent over time. A statistical widening of the gap in preterm birth and smoking rates was found with preterm birth demonstrating a relative increase of 51% over this period.

**Conclusions:**

The comprehensive database from a large urban hospital allowed a thorough examination of outcomes and contributing factors. The gap between both cohorts remains static in several areas but in some cases worsened. Alternative models for delivering care to Indigenous women and their babies have shown improved outcomes, including preterm birth, though not all have been sustained over time and none are available Australia-wide. New models of care, which recognise the heterogeneity of Indigenous communities, incorporate a multiagency approach, and are set within a research framework, are urgently needed.

## Background

Indigenous Australians are one of the most “linguistically and culturally diverse populations in the world” [1] p.3, representing approximately 2.5% of the total Australian population. They also continue to endure widespread and disproportionate disadvantage compared to non-Indigenous Australians on indices of educational attainment, employment, and ill health [2]. Differences in reproductive health outcomes are widely acknowledged with consistent reporting of higher maternal and perinatal morbidity and mortality rates for Indigenous Australians: maternal mortality (5.3 times greater) [3]; low birth weight infants (12.3% vs. 5.9%); preterm births (13.3% vs. 8.0%); perinatal deaths (17.3 vs. 9.7 per 1,000) [4] and infant mortality rate (IMR) (9.6 vs. 4.3 per 1,000) [5]. Life expectancy and access to health care is also considerably worse compared to Indigenous populations in similar countries including Aotearoa/New Zealand (Maori), United States of America (USA) (Indian), and Canada (Inuit) [6-8].

In an effort to address these disparities and reduce the gap in health outcomes between Indigenous and non-Indigenous Australians, the Council of Australian Governments has identified a number of strategic areas for action including: improved antenatal care provision, reducing pregnancy-related alcohol and cigarette consumption, rates of low birth weight (LBW) infants and teenage pregnancy and births, and addressing the causes of maternal mortality and early childhood hospitalisations [7]. The most recent Health Performance Framework Report, on progress against these and other indicators, confirms that despite improvements in some areas (e.g. 34% decline in perinatal mortality between 1999–2008), the initiatives associated with the ‘Close the Gap’ campaign have not made as much progress as anticipated [5]. Whilst selected indicators for Indigenous health generally have shown sustained improvement over recent decades [9], change has not kept pace with improvements noted for non-Indigenous Australians and, hence, the relative gap between the two has actually increased [10]. Differences in outcomes between, and amongst, jurisdictions are evident and do not necessarily follow national trends. For example, trend data on IMR from Western Australia (WA) demonstrated an increase in relative risk (RR) from 3.0 (CI 2.5-3.6) in 1980–84 to 4.4 (CI 3.5-5.5) in 1998–2001 due mostly to a drop in non-Indigenous IMR [11]. The WA data found higher IMR in women under 15 years (29.1), in male infants (22.3), to mothers living in remote areas (23.5), in preterm babies (440.6-29.9), in multiple births (54.7) and to women having ≥5 previous births (28.1) [11]. Additionally, the same study reported a higher relative risk in women age 35–39 years (4.4), in term infants (4.0), in normal birth weight infants (2500-4990 gms) (4.2), and in the postneonatal period (28 days to 12 months) (5.0) [11].

The quality of maternity care provided to Aboriginal women has been highlighted as concerning, and indeed, has been identified as a contributory factor in poor outcomes [12-15]. Findings from a sample of non-Indigenous women at risk of preterm birth identified important issues such as miscommunication and uncaring staff behaviours that negatively influenced care uptake [16], with studies that have focused specifically on the experiences of Indigenous Australians echoing these results [12,13,15]. An added concern in this respect is the tendency to treat Indigenous Australians as an homogenous group rather than as discrete populations with distinctive needs, which may inadvertently contribute to worsening disparities in perinatal outcomes [17]. Targeted models of antenatal care have been developed to address these barriers, with evaluations showing improvements in clinic attendance, screening and treatment (e.g. sexually transmitted and urinary infections) uptake, immunisation rates, mean birth weight, and reduced rates of preterm birth [18-21]. However, none of these programs are available to all childbearing Indigenous women living in Australia.

This paper comments on changes over a 12-year period in selected MIH indicators between Indigenous and non-Indigenous mothers who attended the public facility of a large tertiary maternity hospital in an urban area of South East Queensland, with around 5000 public births per year. The hospital is a referral hospital with a fetal medicine unit and the highest level neonatal nursery taking referrals from across the state. However, it is also the local hospital for a large number of Indigenous women living in the catchment area. The models of maternity care available include hospital and community based antenatal clinics with midwives and medical staff, GP shared care, specialist maternity clinics (e.g. Aboriginal and Torres Strait Islander, Women from refugee backgrounds, Young Women’s Clinic), and community based midwifery group practices offering caseload midwifery care. Allied health referral is commonly used for social work, psychology, mental health, dietetics, and physiotherapy. Data analysis was performed as a component of an evaluation of a specialist collaborative antenatal service targeting Indigenous women or women whose partner’s identified as Indigenous [22].

## Methods

Routinely collected data from 45,216 births (including multiple births) between the years 1998–2009 were analysed; 1,523 births were to Indigenous mothers and 43,693 to non-Indigenous mothers. We excluded data from women (and their infants) transferred in from other areas.

Data were extracted from two hospital obstetric databases: the Obstetric Clinical Reporting System (Clinical Reporting Systems Pty Ltd, Castle Hill, New South Wales, Australia), and MatriX (Meridian Health Informatics, Surry Hills, New South Wales, Australia). For the purpose of this analysis, the term ‘Indigenous’ is defined as women who self-identified as either ‘Aboriginal’, ‘Aboriginal and Torres Strait Islander’ or ‘Torres Strait Islander’. A total of 3.4% women identified as Indigenous, slightly lower than the 3.8% reported nationally [4]. The non-Indigenous cohort comprised primarily Caucasian/European (76.5%) and Asian (12.4%) women. The following ‘Closing the Gap’ indicators [23] were investigated; teenage births (defined as all births to mothers aged less than 20), preterm birth (< 37 weeks), low birth weight (< 2500 g), and smoking (of any frequency, as recorded at first antenatal hospital visit). Additional outcomes included perinatal mortality defined as stillbirth occurring after 20 weeks gestation or greater than 400 grams, or neonatal death occurring within 28 days of birth, very preterm birth (< 32 weeks) and small for gestational age (SGA) infants, defined as < 10^th^ centile on a population-based standard [24].

Additional variables, which included maternal age, self-reported pre-pregnancy weight (subsequently used to calculate body mass index [BMI]), education level, marital status, pre-existing medical conditions, selected social and lifestyle indicators (routinely collected at the booking visit to determine the basis for referral), and labour and birth indicators, were compared between the Indigenous and non-Indigenous cohorts.

All variables are reported as the number and proportion within the two cohorts, with the difference in proportions calculated as well as the corresponding 95% confidence interval (CI) of the difference. Where missing data are apparent, the denominator used is the total non-missing entries. Trend analysis was undertaken with the 12 years of data amalgamated into triennia due to the small number of outcomes in the Indigenous cohort. Logistic regression was carried out to assess trends, using the independent variables of ethnicity (Indigenous versus non-Indigenous) and triennia, with an interaction term also included to investigate whether the rate of each outcome had changed over time between the two cohorts. Additional multivariate analysis was carried out for key indicators using logistic regression to adjust for important confounders. Bivariate and multivariate odds ratios (ORs) are reported, along with 95% CIs. Statistical significance was set at 0.05. Data were collated and analysed using StataSE Version 10 (StataCorp, College Station, Texas, USA), with graphical interpretation of the results generated using Microsoft Office Excel 2002 (Microsoft Corporation, Redmond, Washington, USA). This study was approved by the Hospital HREC Health Services Human Research Ethics Committee.

## Results

Indigenous women birth at significantly younger ages compared to their non-Indigenous counterparts. Almost half of the Indigenous women who birthed at the MMH were under the age of 25 years (46.0%) compared to 25.6% of non-Indigenous women; 18.3% of Indigenous women were teenagers (< 20 years of age). This represents a 12.7% difference in teenage births (< 20 years of age) when compared to non-Indigenous mothers (18.3% vs. 5.6%; 95% CI 10.7, 14.6%). Differences in BMI were mainly observed with respect to the normal BMI weight category (18.5- < 25) (45.3% vs. 55.6%) favouring the non-Indigenous cohort; more Indigenous women were also categorised as obese: 20.8% vs. 15.0%. However, as over 22% of missing data were noted for the Indigenous cohort, this result should be viewed with caution. Indigenous women were over-represented in the lower education categories and under-represented in tertiary education: ≤ Grade 10 (21.9% difference, 95% CI 19.2, 24.6%); Grade 11 and 12 (1.8% difference, 95% CI −0.8, 4.4%) and tertiary education (20.0% difference, 95% CI −21.8, -18.2%). A considerably higher percentage of Indigenous women were either single or never married (40.5% vs. 13.5%, difference 26.9%, 95% CI 24.4, 29.5%). No statistically significant differences in rates of pre-existing medical conditions were observed between the two cohorts. Significant differences were observed, however, between several psychosocial indicators routinely collected during pregnancy, which suggested that women might benefit from additional support during the maternity period (Table 1). Caution with interpretation of some figures is necessary as data were only available from 2007–09 for some variables.

**Table 1:** Key social indicators by Indigenous status

**Social indicators**	**Indigenous%**			**Non-Indigenous %**			**Difference**		
	**N**	**n**	**%**	**N**	**n**	**%**	**%**	**95%**	**CI**
Domestic Violence (afraid for physical safety)^a^	301	13	4.3	7,089	149	2.1	2.2	−0.1	4.5
Domestic Violence (emotional abuse)^a^	298	16	5.4	7,089	230	3.2	2.1	−0.5	4.7
Smoking at 1st visit^b^	1,495	733	49.0	42,793	8,174	19.1	29.9	27.4	32.5*
Current Cannabis use at 1st visit^b^	1,137	72	6.3	33,279	564	1.7	4.6	3.2	6.1*
Alcohol consumption during pregnancy^a^	335	31	9.3	9,165	672	7.3	1.9	−1.2	5.1
DOCS^a^	342	42	12.3	9,117	239	2.6	9.7	6.2	13.2*
EDS >14^a^	155	14	9.0	7,989	421	5.3	3.8	−0.8	8.3

* Statistically significant; CI = Confidence Interval; DOCS = Department of Child Safety; EDS = Edinburgh Depression Scale.

^a^ Data May 2007–December 2009 only. Total Indigenous pregnancies n = 32, total non-Indigenous pregnancies n = 9,516.

^b^ Data 1998–2009. Total Indigenous pregnancies n = 1,499, total non-Indigenous pregnancies n = 42,810.

Indigenous women were statistically more likely to have spontaneous onset of labour, opioids for pain relief in labour, a non-instrumental vaginal birth, and less likely to have an epidural for pain relief in labour, experience an assisted birth, perineal trauma or caesarean section (Table 2).

**Table 2:** Key maternal and neonatal indicators by Indigenous status

**Indicator**	**Definition**	**Indigenous**			**Non-Indigenous**			**Difference**		
		**N**	**n**	**%**	**N**	**n**	**%**	**%**	**95%**	**CI**
Gestation at birth^a^	< 32 weeks	1,499	51	3.4	42,810	892	2.1	1.3	0.4	2.2*
	< 37 weeks	1,499	191	12.7	42,810	3,787	8.9	3.9	2.2	5.6*
Onset of labour^a^	Spontaneous	1,499	1,027	68.5	42,805	27,154	63.5	5.1	2.7	7.5*
	Induced		298	19.9		9,931	23.2	−3.3	−5.4	−1.3*
	No labour - CS		174	11.6		5,720	13.4	−1.8	−3.4	−0.1*
Analgesia^a^	Inhalational	1,380	649	47.0	39,365	18,263	46.4	0.6	−2.0	3.3
	Opioid	1,380	368	26.7	39,365	9,031	22.9	3.7	1.4	6.1*
	Epidural	1,380	287	20.8	39,365	10,754	27.3	−6.5	−8.7	−4.3*
Five min Apgar score^b^	< 7	1,520	36	2.4	43,612	917	2.1	0.3	−0.5	1.0
Method of birth^b^	Non-instrumental vaginal	1,516	1,039	68.5	43,460	27,079	62.3	6.2	3.8	8.6*
	Forceps		16	1.1		989	2.3	−1.2	−1.8	−0.7*
	Vacuum		69	4.6		3,102	7.1	−2.6	−3.7	−1.5*
	Caesarean section		392	25.9		12,290	28.3	−2.4	−4.7	−0.2*
VBAC^a^	Vaginal birth, parity 1, previous CS; singleton cephalic term	52	8	15.4	2,150	457	21.3	−5.9	−15.8	4.1
Perineal trauma^c^	Intact or 1^st^ degree tear	1,320	1,146	86.8	37,005	28,072	75.9	11.0	9.1	12.8*
	2^nd^ degree tear		153	11.6		6,072	16.4	−7.2	−8.8	−5.6*
	3^rd^ or 4^th^ degree tear		52	3.9		2,861	7.7	−3.8	−4.9	−2.7*
	Episiotomy	1,319	73	5.5	36,995	3,705	10.0	−4.5	−5.8	−3.2*
SGA^b^	< 10^th^ centile	1,476	207	14.0	41,934	4,115	9.8	4.2	2.4	6.0*
Low birth weight^b^	< 2500 g	1,523	201	13.2	43,691	3,586	8.2	5.0	3.3	6.7*
Perinatal outcome^b^	Stillbirth	1,523	12	0.8	43,693	298	0.7	0.1	−0.3	0.6
	Neonatal death		13	0.9		224	0.5	0.3	−0.1	0.8
	(Perinatal deaths)		25	1.6		522	1.2	0.4	−0.2	0.1
	Survived		1,498	98.4		43,171	98.8			
Admission to NCCU^b^		1,523	280	18.4	43,693	6,004	13.7	4.6	2.7	6.6*
Length of stay	Maternal < 3 days^a^	1,492	985	66.0	42,655	24,892	58.4	7.7	5.2	10.1*
	Neonate in NCCU > 7 days^b^	1,523	41	2.7	43,693	683	1.6	1.1	0.3	2.0*
Feeding at discharge^b^	Breast milk	1,385	959	69.2	40,881	31,192	76.3	−7.1	−9.5	−4.6*
	Breast & formula		92	6.6		3,688	9.0	−2.4	−3.7	−1.0*
	Formula		315	22.7		5,730	14.0	8.7	6.5	11.0*
	Gavage		19	1.4		218	0.5	0.8	0.2	1.5
	Other		0	0.0		53	0.1	−0.1	−0.2	−0.001*

*Statistically significant. CS caesarean section; VBAC vaginal birth after caesarean; NCCU Neonatal Critical Care Unit; SGA small for gestational age.

^a^ Data 1998–2009. Total Indigenous pregnancies n = 1,499, total non-Indigenous pregnancies n = 42,810.

^b^ Data 1998–2009. Total Indigenous births n = 1,523, total non-Indigenous births n = 43,693.

^c^ Data excluding CS with no labour. Total Indigenous pregnancies n = 1,325, total non-Indigenous births n = 37,085.

Compared with babies born to non-Indigenous mothers, Indigenous babies born to Indigenous women were statistically more likely to be: born preterm, of low birth weight, admitted to the NCCU, and if admitted more likely to be admitted for more than seven days, and formula feeding on discharge from hospital. Mothers of Indigenous infants, however, were more likely to stay less than three days in hospital following birth.

### Teenage births

**Figure 1:**
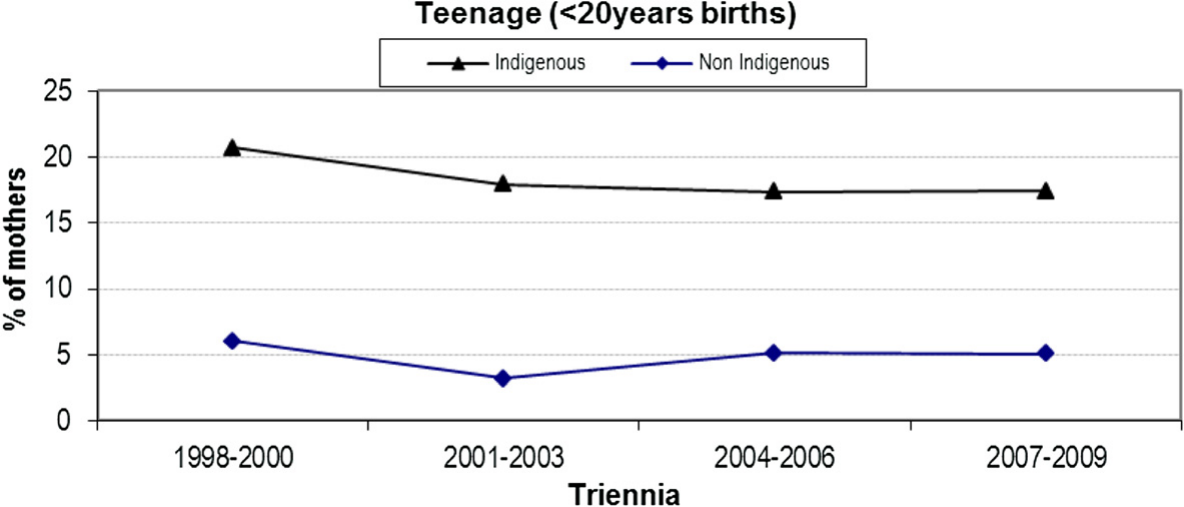
Trend analysis of teenage births by triennia and Indigenous status.

The difference in the rate of teenage births (< 20 years of age) between Indigenous and non-Indigenous women has remained relatively unchanged over the last eight years (Figure 1). Although 2001–2003 and 2004–2006 saw a slight drop from the previous triennium (1998–2000), the rates have since remained steady. The percentage difference over the last three triennia remains at approximately 13% with no interaction evident between Indigenous status and time (p = 0.90). Both cohorts have seen a slight but sustained drop in rates over time.

### Smoking

**Figure 2:**
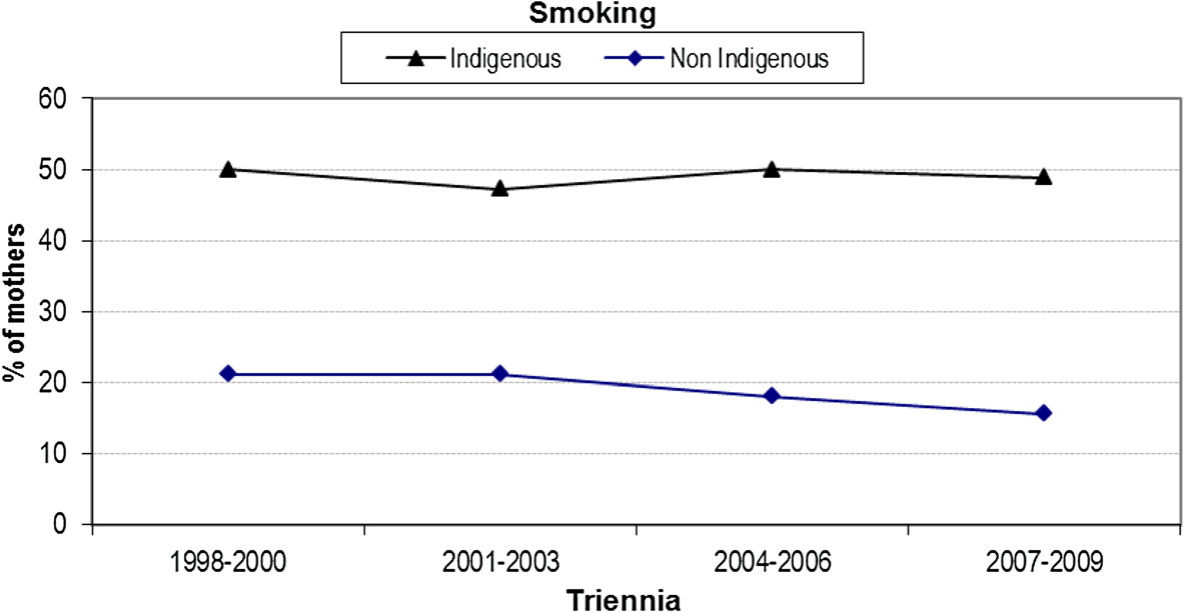
Trend analysis of smoking status at first antenatal (booking) visit by triennia and Indigenous status.

Bivariate analysis revealed a statistically significant difference between Indigenous and non-Indigenous smoking status at the antenatal hospital booking visit (community based antenatal care is provided and women are referred for a hospital booking at approximately 17–24 weeks gestation) (48.9% vs. 19.2%; difference 30.0%, 95% CI 27.2, 32.3%). A significant interaction was observed between Indigenous status and year, indicating a downward trend amongst non-Indigenous women that was not observed after the first triennia in the Indigenous cohort (p = 0.01) (Figure 2).

### Small for gestational age

**Figure 3:**
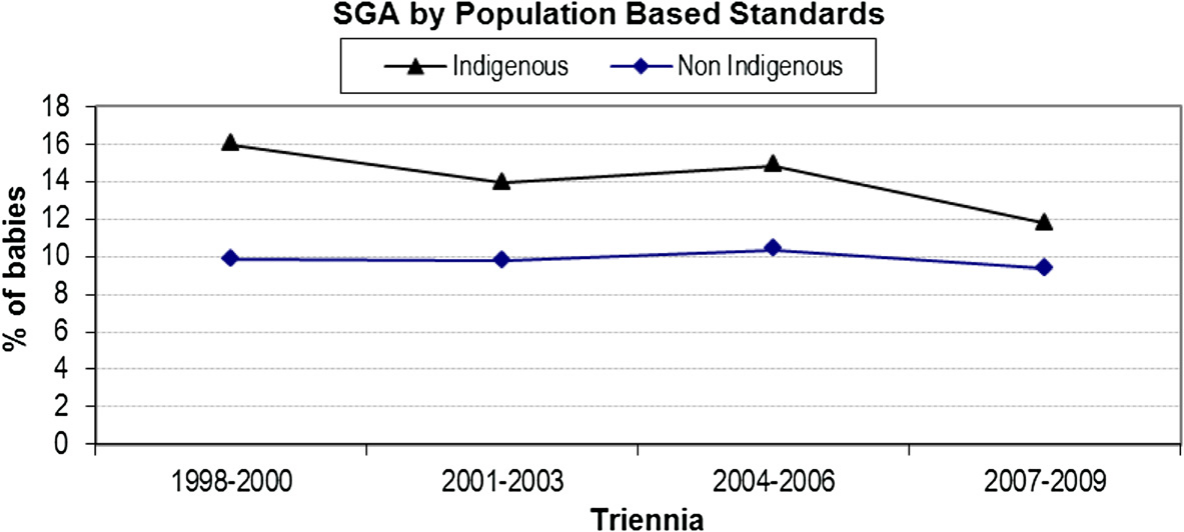
Trend analysis of small for gestational age by population based standards by triennia and Indigenous status.

There was no significant interaction (p = 0.20) between Indigenous status and year of birth for SGA infants (Figure 3). Whilst the rate of SGA births to non-Indigenous women remained steady, there was some evidence of a decrease for Indigenous women.

### Preterm birth

**Figure 4:**
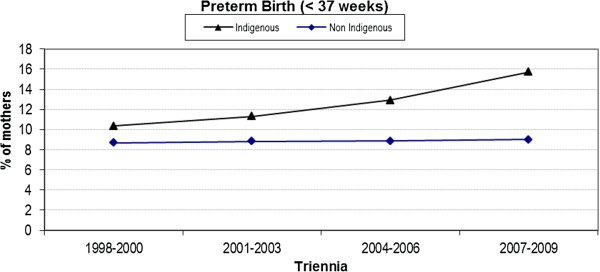
Trend analysis of preterm birth < 37 weeks by triennia and Indigenous status.

The difference in the rate of preterm birth (< 37 weeks) between the two cohorts was very small in the 1998–2000 (first) triennium, however, this difference has gradually increased resulting in a percentage difference of nearly 7% (15.7% versus 9.0%) in 2007–2009 (Figure 4); this observation was confirmed via a statistically significant interaction term (p = 0.04).

### Very preterm birth

**Figure 5:**
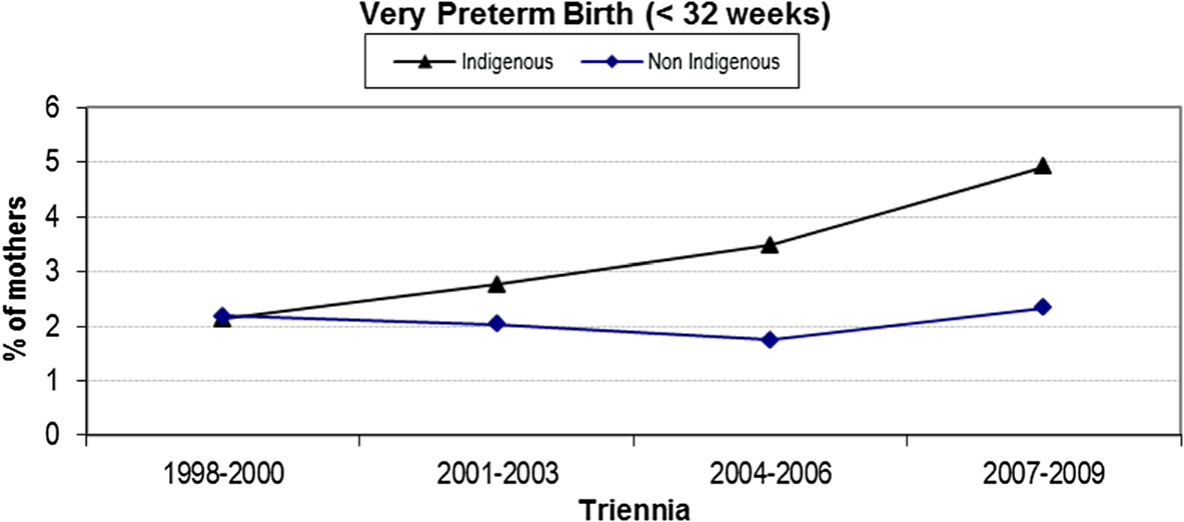
Trend analysis of very preterm birth < 32 weeks by triennia and Indigenous status.

Similarly, the difference in the rate of very preterm birth (< 32 weeks) between the two cohorts was initially relatively small but has since increased to a difference of nearly 3% (4.9% versus 2.3%) in the latest triennium (Figure 5). Again, significant interaction between Indigenous status and triennia of birth was noted, indicating that the rate of very preterm birth has widened over time (p = 0.04).

### Low birth weight

**Figure 6:**
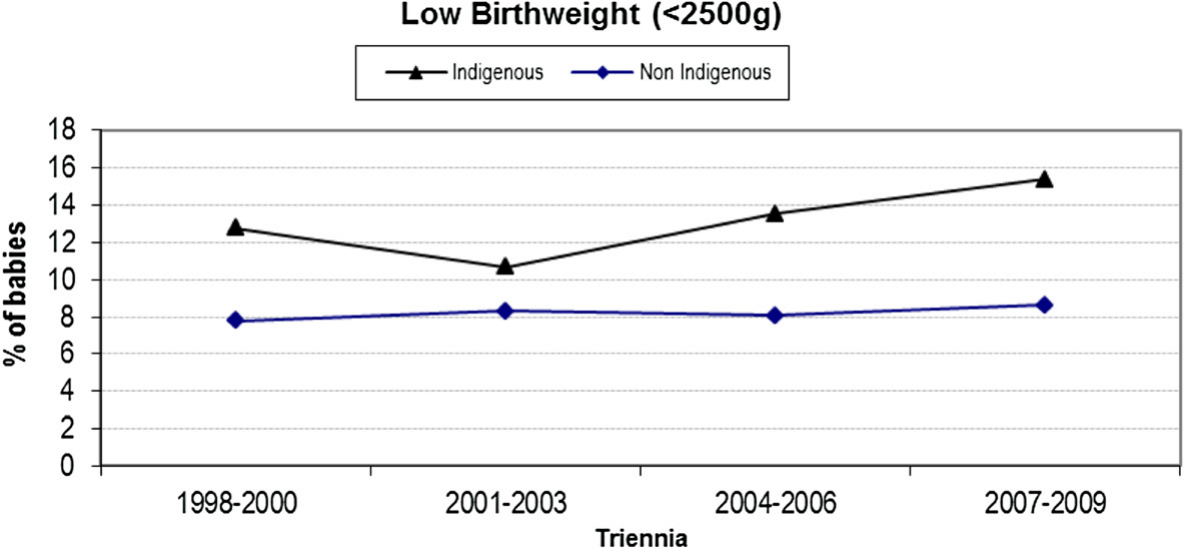
Trend analysis of low birth weight infants < 2500 g by triennia and Indigenous status.

Differences in LBW (< 2500 g) rates between the cohorts has remained relatively constant over the 12 years of the study period (Figure 6) with an average difference of 5%. As such, interaction between Indigenous status and year of birth is not significant (p = 0.30).

### Perinatal mortality

**Figure 7:**
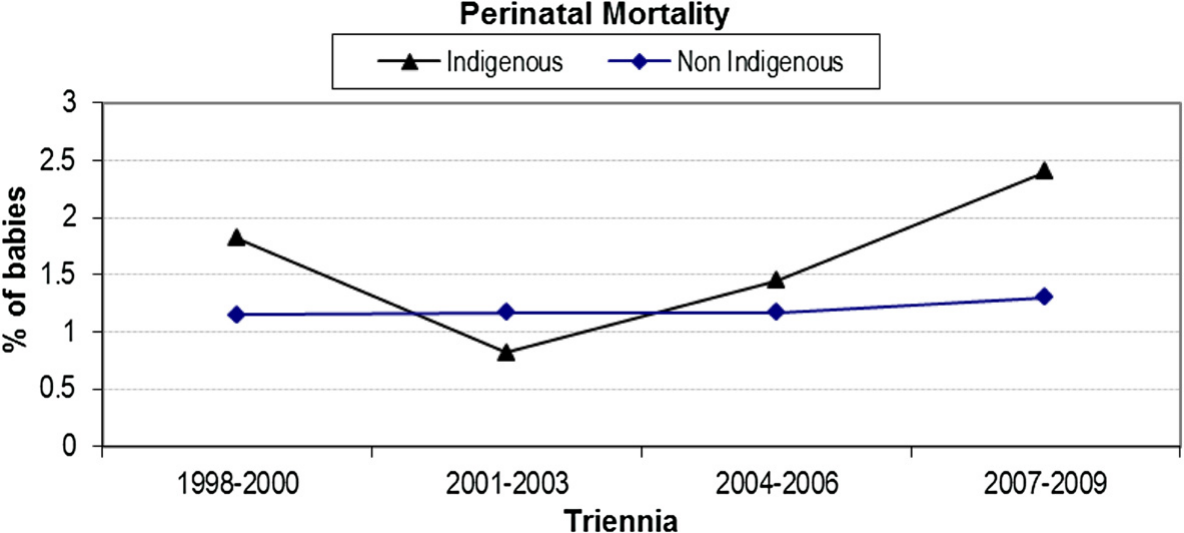
Trend analysis of perinatal mortality by triennia and Indigenous status.

There was no significant interaction (p = 0.49) between Indigenous status and triennia of birth in relation to perinatal mortality (Figure 7). The increase in the last two triennia may be accounted for by stillbirths occurring during this period; the proportion of stillbirths of all perinatal deaths has increased from 50.3% in 1998–2000 to 59.9% in 2007–2009. Caution should be exercised when reading this analysis, however, as percentages are low.

#### Multivariate analysis

Table 3 presents a comparative analysis between bivariate and multivariate analyses of key indicators previously described. Preterm birth, low birth weight, and teenage birth rate remained statistically significant when adjusted for age, parity, BMI, smoking, gestational diabetes, antepartum haemorrhage, pregnancy induced hypertension and socio-economic status (using Socio Economic Indexes For Areas [25]) in the multivariate analysis. However, very preterm birth (< 32 weeks) although statistically significant in the bivariate analysis, was not statistically significant when adjusted in the multivariate.

**Table 3:** Indigenous vs. non-Indigenous bivariate and multivariate analysis of key indicators

**Outcome**	**Univariate**				**Multivariate**			
	**OR**	**95%**	**CI**	**P**	**aOR**	**95%**	**CI**	**P**
Preterm < 37 weeks	1.42	1.19	1.70	0.001*	1.21	1.01	1.46	0.04*
Preterm < 32 weeks	1.62	1.16	2.26	0.004*	1.26	0.89	1.78	0.19
Low birth weight	1.66	1.39	1.99	0.001*	1.31	1.09	1.58	0.01*
Perinatal mortality	1.51	0.94	2.42	0.088	1.22	0.75	1.99	0.42
Teenage births#	4.12	3.53	4.81	0.001*	4.24	3.55	5.07	<0.001*

OR odds ratio; aOR adjusted odds ratio; CI confidence interval; p p-value; # adjusted for parity, year of birth, BMI, smoking, gestational diabetes, APH, PIH and SEIFA. All other variables adjusted for age, year of delivery, parity, BMI, smoking, gestational diabetes, APH, PIH and SEIFA. * Statistically significant.

## Discussion

Women of childbearing age and mothers of young children constitute a significant proportion of Indigenous Australians. Given the substantial and longstanding evidence linking economic disadvantage with negative intrauterine and early infancy events, and indeed with poorer health over the life-course [26-29], and given the pivotal role of mothers as primary carers in family settings [30,31], the importance of prioritising their health cannot be over-emphasised [32]. A comprehensive database, from a large urban tertiary hospital in Queensland with transfers in excluded from analysis, has allowed a more thorough examination of data controlling for possible confounders, than can be performed at a national or jurisdictional level. We found significant differences between the cohorts for many outcomes although we also observed relatively stable rates for smoking at booking, low birth weight, small for gestational age babies, and perinatal morality over the past decade for Indigenous women. However, a statistical increase and a widening of the gap in preterm birth rates between the Indigenous and non-Indigenous cohort was found, together with a widening of the gap in smoking rates driven by a decrease in non-Indigenous rates.

Both cohorts experienced a non-significant decline in teenage pregnancy over four triennia although the percentage difference remained approximately 12%. The fall in teenage births to Indigenous women (1998: 21.4% to 2009: 18.2%) is not reflected in the Queensland state-wide data where the rate of teenage births remained almost unchanged (2000: 19.3% to 2009: 19.4%). This may reflect increased access to education and/or contraception in the urban area. Following multivariate analysis, Indigenous women were statistically more likely to experience teenage birth than their non-Indigenous counterparts (aOR 4.24, 95% CI 3.55, 5.07). Hence the need to carefully consider how services might be designed and delivered to this group of women, to increase acceptability and improve clinical outcomes. A literature review on models of maternity care for young women (aged 21 years and under) suggests group antenatal care is associated with higher antenatal attendance, lower preterm birth and higher breastfeeding initiation, and a multi-disciplinary young women’s clinic may also improve antenatal visit attendance and reduce preterm birth [33]. The review found no studies that examined the acceptability or impact of midwifery group practice models of care, known to benefit women generally [34], and particularly Indigenous women [35] and teenage women.

Despite differences in the content and frequency of data collection for MIH outcomes across Australia, national data confirms that approximately 50% of Indigenous mothers smoked tobacco at some stage of their pregnancy [36]; findings from our evaluation reflect this with trend data showing an increasing gap over time in women smoking at their first (booking) visit. Although smoking during pregnancy is associated with a higher prevalence of preterm birth (approximately 40%), and almost 100% higher proportion of low birth weight infants, national data [36] suggests that rates of preterm births, LBW infants, and perinatal deaths were higher for infants born to Indigenous mothers regardless of pregnancy-related smoking status. Strategies to date to address smoking amongst pregnancy in Indigenous women have been relatively unsuccessful, perhaps not least because research suggests that social context is an important factor with smoking providing a sense of belonging and identity, and group membership [37]. Smoking cessation interventions in pregnancy, however, are known to make a difference to low birth weight and preterm birth in the general population of childbearing women [38] with a recent survey of Aboriginal women reporting 21% quit in pregnancy with 46% reducing their intake. Whilst care providers understood the risks and regularly assessed smoking in pregnancy, not all had good knowledge about smoking cessation initiatives [39]. Our study highlighted problems with data collection throughout pregnancy, making ongoing assessment of smoking status and the timely delivery of smoking cessation strategies difficult to report.

In our study, Indigenous women were statistically more at risk of psychosocial and emotional challenges during pregnancy, including: domestic violence, cannabis use and contact with the Department of Child Safety. Although the hospital-based Indigenous liaison team provides support to Indigenous women accessing the antenatal clinic, the lack of a dedicated social worker providing continuity, was identified as problematic [40]. Evaluation data identified that Indigenous women were less likely to identify isolation as a concern for them [22], perhaps due to the extended family support that is known to be a feature of Indigenous life. Studies have reported a family-centred approach to health and education [41,42] with the support of family seen to be of significant benefit to the pregnant woman in a variety of ways, for example, psychosocial support, child care, role modelling by elders, cultural education and support of cultural and community values [43].

Indigenous women experienced fewer interventions in birth with higher rates of spontaneous onset of labour and non-instrumental vaginal birth, and lower rates of epidurals for pain relief in labour, assisted births (both forceps and vacuum extraction), perineal trauma and caesarean sections. Despite these impressive maternal outcomes the poorer outcomes for babies are concerning. National perinatal data for the years 1991–2008 reported that Indigenous mothers are twice as likely to give birth to LBW babies compared to non-Indigenous mothers, with the overall rate of LBW infants having increased by 13% over this period [36]. Our data in this area reflects national trends although our rates of LBW Indigenous infants increased less dramatically. Multivariate analysis, , however, showed a significant difference between the cohorts (OR 1.31, 95% CI 1.09, 1.58) after adjustment for age, year of delivery, parity, BMI, smoking, gestational diabetes, APH, PIH and SEIFA.

Preterm (< 37 weeks) birth rates are increasing for all Australian women with a study of more than two million pregnancies reporting an almost 20% increase in the proportion of low-risk women having a preterm birth over the 10 years from 1994, with a 12% rise in preterm birth overall [44]. National data [45] reveals that Indigenous women were significantly more likely to give birth before 37 weeks with Queensland data also showing a significant difference (12.5% vs. 7.5%; OR 1.71: 95% CI 1.65, 1.77) [46]. This finding is mirrored in our multivariate analysis (OR 1.21: 95% CI 1.01, 1.46) which adjusted for well known confounders. The rate of preterm birth for Indigenous women in our dataset increased by 5.0% (a relative increase over the time period of 51.0%) compared with the smaller increase of less than 1% (8.7% to 9.0%) observed for non-Indigenous women, and in opposition to the state-wide data which showed a decrease from 13.1% to 11.6% over similar years (2000–09). Three percent of the absolute increase in our data is accounted for in the <32 weeks gestational age bracket with 4.0% in the 32–37 week bracket (data not shown). The reasons our data differs from state data are unknown, and were unexpected, as we had excluded all transfers in (transfers can be from across the state or even interstate usually for women requiring the highest level of tertiary care). However, our service does not exclude any eligible woman who live in the hospital metropolitan area and who self present requesting care, and mapping the postcodes of service users’ places of residence against the 2006 census (suburb and postcode) identified a number of women self-referring to the hospital from outside the local catchment area [22]. It is possible that the differences in our data are due to women with pre-existing risk factors self-selecting this tertiary hospital because of the specialist collaborative Indigenous maternity provision, or being referred by external providers. Alternatively, we may have better ascertainment of Indigenous status than state-wide data, or our findings may reflect a genuine increase in preterm rates for Indigenous women living in our area.

Preterm birth is a leading cause of perinatal mortality, serious neonatal morbidity and moderate to severe childhood disability [44,47-49]. Research in Queensland [50] and Western Australia [11] identified that the majority of Indigenous perinatal deaths are due to antenatal factors with significantly more potentially preventable deaths due to infection, preterm birth and sudden infant death syndrome. These are all amenable to targeted interventions with Queensland results [50] recommending primary health care initiatives to reduce the prevalence of low birth weight and preterm birth; and a public health approach inclusive of a domestic violence focus. Preterm birth correlates strongly with poverty and socio-economic status [51], maternal psychosocial stress [52], smoking in pregnancy [53], limited maternal education and young maternal age [47]. All of these risk factors were statistically more likely to be present in our Indigenous cohort and when they were controlled for in multivariate analysis a difference in preterm birth and low birth weight infants remained. Additionally, modifiable risk factors for stillbirth such as overweight, obesity and smoking were significantly higher in the Indigenous cohort and have been identified as priority areas for stillbirth prevention in high-income countries [54].

## Conclusion

It is clear that more should, and could, be done to prevent poor outcomes. Further redesign of services is urgently needed to ‘close the gap’ in poor MIH outcomes between Indigenous and non-Indigenous Australians. A focus on culturally responsive care that incorporates strategies for targeting modifiable risk factors in the early antenatal period, with interventions that span the continuum of care from preconception to infancy, is essential. This should be done by building on strategies that have been shown to make a difference [55,56]. Our data indicates that targeting early preterm birth will be important to address our worrying finding about the increase in very preterm births (< 32 weeks). The differences in socio-economic outcomes and high rates of smoking in pregnancy highlighted the challenges in this area. It was encouraging to see that the rising preterm rates had not resulted in a statistically significant increase in PMR (trend), however it is possible this is due to the small numbers as the data have shown an increase in this rate over the time period. A multiagency response would assist in ensuring all possible resources can be cohesively directed towards remedying one of Australia’s greatest, and most persistent, challenges: that of improving health outcomes for Indigenous mothers and their infants.

## Competing interests

There are no competing interests, financial or otherwise, for any of the authors.

## Authors’ contributions

SK conceived of the study, and participated in the design and coordination. SK also drafted the manuscript. HS assisted in the coordination of the project, and contributed to the drafting of the manuscript. RM conducted the quantitative analysis and helped draft the manuscript. MK helped to design the study, collect the data and reviewed the manuscript. KG coordinated the quantitative analysis and helped draft the manuscript. All authors read and approved the final manuscript.

## Pre-publication history

The pre-publication history for this paper can be accessed here:

http://www.biomedcentral.com/1471-2393/13/167/prepub
